# Preference for breast cancer screening in Chinese women: Discrete choice experiment

**DOI:** 10.1002/cam4.70013

**Published:** 2025-10-30

**Authors:** Shuning Wu, Jianhao Du, Yifei Wang, Dawei Peng, Xinyang Ma, Jiali Chen, Jie Li, Gezi Li, Xing Li, Jun Zhang, Wai‐Kit Ming

**Affiliations:** ^1^ Thyroid and Breast Surgical Department Shenzhen Qianhai Shekou Free Trade Zone Hospital Shenzhen China; ^2^ School of Medicine Jinan University Guangzhou China; ^3^ Department of Infectious Diseases and Public Health Jockey Club College of Veterinary Medicine and Life Sciences, City University of Hong Kong Hong Kong China

**Keywords:** breast cancer, breast cancer screening, COVID‐19, discrete choice experiment, tertiary prevention

## Abstract

**Background:**

Breast cancer is a common tumor in women, which significantly impacts their health and quality of life. The incidence of breast cancer has been increasing globally.

**Objective:**

This study aimed to investigate the preferences of Chinese women regarding breast cancer screening (BCS) during the COVID‐19 pandemic and examine how these preferences influence their screening choices.

**Method:**

The online DCE (Discrete Choice Experiment) questionnaire was designed through Sawtooth Lighthouse Studio (9.8.1) with the following selected attributes: acceptable hospital distances, acceptable hospital grades, duration of each screening, main screening method, total acceptable length of screening, and total cost of screening. Data were analyzed with a mixed logistic analysis after the data collection was completed. A latent class analysis was also performed to observe the screening preferences of participants, their willingness to pay, and to infer whether the distribution of preferences between the different parties was statistically significant.

**Results:**

A total of 397 people filled out the questionnaire, and 325 people met the inclusion criteria after being screened by trap questions. Each attribute is important in shaping participants' preferences for BCS provision. People preferred “Ultrasound Screening,” “No side effects,” higher medical reimbursement rates, and lower costs.

**Conclusion:**

These findings provide valuable insights into the thoughts and preferences of participants regarding BCS. Healthcare providers should take these preferences into consideration to improve patient compliance and enhance the effectiveness and safety of clinical care.

## Graphical abstract

This study investigated Chinese women's preferences for breast cancer screening choices during the COVID‐19 pandemic and how these preferences influence their choices. The results indicated that “Ultrasound Screening,” “Day of appointment,” and “No side effects” were the preferred options.

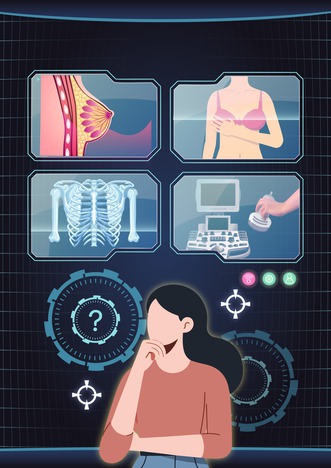

## 1 INTRODUCTION

Breast cancer is a prevalent and significant health concern for women. Annually, approximately 2.1 million new cases are reported globally, making up 25% of all female cancer cases.^1^ The rising burden of breast cancer among Chinese women emphasizes the importance of increasing screening rates.

While the incidence of breast cancer is on the rise, the mortality rate is decreasing due to the efforts of health campaigns, expanded screening programs, and advancements in treatment. Breast cancer screening (BCS) plays a particularly vital role in this regard.^1^, ^2^

In China, BCS is a crucial component of the national basic public health services.^3^ The guidelines advise one to two screenings per year for women over 40 years old at average risk and yearly screening for women at high risk. However, the screening rates in China are relatively low, with only 22.5% in 2013.^4^

The effectiveness of a screening program relies on the active involvement of the public. Discrete choice experiments (DCEs) are a valuable tool for gaining insights into women's decision‐making process regarding BCS.^5^, ^6^ A DCE enables the identification of public preferences and serves as a foundation for implementing strategies that can enhance screening rates. Through the use of various scenarios, a DCE allows participants to express their preferences for different screening attributes such as cost, procedure, and travel time.^7^ Applying DCE to BCS can provide valuable insights into women's preferences for the screening intervention attributes.

However, it is crucial to have an understanding of women's preferences for BCS attributes in China in order to enhance its acceptance and effectiveness. Aligning the screening attributes and levels of provision with the preferences of the target population is important to ensure high participation rates. Although mammography is currently recommended as the primary screening method for women at average risk, it may cause pain and discomfort.^8^

In this study, we assessed women's preferences for BCS attributes using the DCE. By quantitatively understanding the relative importance of these attributes, decision‐makers can make informed choices when designing BCS by selecting meaningful levels of attributes.

## 2 METHODS

### 2.1 Study population

For this study, we selected Chinese women between the ages of 30 and 69 who had resided in Shenzhen for more than 5 years. They were required to independently complete the questionnaire. Data were gathered from September 2020 to June 2021 at community hospitals in Nanshan District, Shenzhen, Guangdong Province. Participants completed the questionnaire by scanning a QR code using their mobile phones and received support from a healthcare professional to ensure accuracy.

### 2.2 Discrete choice experiment

Current guidelines are the basis for the design and analysis of DCE.^9^ Random utility theory is the theoretical basis of DCE.^10^ Unlike other approaches, the DCE method simulates decision‐makers making actual choices in real‐life scenarios. Participants are presented with different hypothetical options that vary in attributes. They then choose their preferred option based on maximizing utility. DCEs are commonly utilized in health economics to tackle health policy issues.

### 2.3 Attribute and attribute levels

Identifying attributes and corresponding levels is the first step in designing a DCE questionnaire. The attributes and levels of this DCE questionnaire were determined by searching the relevant literature as well as by expert assessment. Seven attributes were chosen: screening tools, appointment time, travel time, side effects, medical reimbursement, screening sensitivity, and cost (Table 1).

**TABLE 1:** Attributes and levels.

Attributes	Levels of attributes
Screening methods	
L1	Clinical breast examination
L2	Mammography screening
L3	Ultrasound screening
L4	MRI screening
Appointment length	
L1	Day of appointment
L2	Tomorrow
L3	1 week
Travel time	
L1	30 min
L2	60 min
L3	90 min
L4	120 min
Side effects	
L1	No side effects
L2	Noninvasive for pain
L3	Invasive
L4	Radiation
Medical reimbursement	
L1	0%
L2	50%
L3	90%
Total cost of screening	
L1	100RMB
L2	400RMB
L3	700RMB
L4	1100RMB
L5	1300RMB
Screening sensitivity	
L1	30%
L2	50%
L3	70%
L4	90%

We discussed the feasibility of these attributes with breast cancer specialists at Shenzhen Qianhai Shekou Free Trade Zone Hospital and established the relevant levels of the attributes. In order to ensure the smooth execution of our questionnaire survey, we included five volunteers to participate in a pilot study. The purpose of this pilot study was to test the questionnaire and make any necessary adjustments or refinements before the actual survey.

### 2.4 Study design and questionnaire

The questionnaire is divided into two parts. The first part collects basic demographic information, such as occupation, education, income, marital status, and knowledge of breast cancer. The second part involves answering 10 random‐choice questions and two fixed‐choice questions in a DCE scenario. Each question presents three options: option A, option B, and none. In the random‐choice questions, the attribute level changes with each question, while in the fixed‐choice questions, the attribute level remains the same. Each questionnaire will include two fixed‐content questions (trap questions) designed to assess respondents' understanding of the questionnaire content and the authenticity of their responses. These two questions offer clearly distinguishable options, with one option being more favorable than the other. By analyzing whether participants consistently select the more favorable option, the effectiveness of the questionnaire data can be determined. Table 2 provides an example of a DCE random‐choice question.

**TABLE 2:** An example of DCE random‐choice question.

Attributes	Option A	Option B	Option C
Screening methods	Mammography screening	Ultrasound screening	
Appointment length	Tomorrow	1 week	
Travel time	90 min	120 min	
Side effects	Noninvasive for pain	Invasive	
Medical reimbursement	0%	50%	
Cost	400RMB	700RMB	
Screening sensitivity	50%	70%	
Which scenario would you choose?	Select	Select	Select

It is not possible to present all the scenarios of attribute level permutations in one questionnaire, there are 11,520 (4 × 3 × 4 × 4 × 4 × 3 × 5 × 4) scenarios of attribute level permutations in this study. Therefore, we utilize fractional factorial designs generated through Sawtooth software to ensure both the orthogonal properties and level balance within the design. By employing fractional factorial designs, we aim to achieve a balanced representation of each level of the factors or attributes under investigation, minimizing any potential bias or confounding effects arising from uneven distribution. This level balance allows us to enhance the internal validity of our study and accurately attribute observed effects to the specific factors or attributes being studied.^11^, ^12^ We generated 100 versions of the questionnaire using the fractional factorial design module in Sawtooth software and allocated the questionnaires to participants using the software's complete random allocation feature to ensure fairness and reduce bias.

### 2.5 Sample size

We determined the minimum sample size based on Johnson and Orme's empirical rule formula, considering the interaction between medical reimbursement and expenses. We established a minimum sample size of 375 for this study.

### 2.6 Statistical analysis

The collected data were analyzed using a conditional logistic regression model.^13^ The choice was considered as the dependent variable, while the attribute level was taken into account as the covariate. We used conditional logistic regression models to analyze the relationship between selection and individual attribute levels. This allowed us to statistically infer participants' preference weights for each attribute and level in the questionnaire. The coefficients generated by the regression analysis indicate the direction of preference for each attribute, while the magnitude of the coefficients estimates the relative importance of the attributes.

The data underwent analysis using a mixed logit model, which is grounded in the principles of random utility theory. In this framework, the utility associated with respondent *i*'s selection of alternative *j* within choice set *t* is expressed as

$$U_{\mathrm{ijt}}=X_{\mathrm{ijt}}\beta_{i}+\varepsilon_{\mathrm{ijt}};i=1,\ldots ,n;j=1,2,3;t=1,\ldots ,100,$$

where *X*_*ijt*_ is a vector of variables representing the alternative specific constant and attributes of alternative *j* and *β*_*i*_ is a vector of random coefficients assumed to be uncorrelated and normally distributed except for the coefficients of the cost and effectiveness attributes, which were assumed to be distributed log‐normally.

Additionally, we calculated marginal rates of substitution and costs, which represent the ratio of the coefficients, in order to examine the relative importance of the attributes.

The latent class model is a statistical technique used to identify and evaluate the variation in preferences among different subgroups within a population. The Bayesian information criterion is used to determine the optimum number of categories for LCA.^14^ The WTP (willingness to pay) value is calculated by the ratio of the random coefficients of the attributes (the denominator is the cost attribute coefficient). WTP refers to the amount of money someone is willing to pay in order to enhance a specific attribute. WTP values provide insight into an individual's level of preference for that particular attribute. All statistical analyses related to the DCE in this study, including choice modeling, utility weight estimation, and calculation of percentages, were conducted using Sawtooth Lighthouse Studio (SSI Web version 9.4.0; Sawtooth Software Inc.). This software was employed exclusively for the DCE analysis presented in this paper.

## 3 RESULTS

### 3.1 Descriptive statistics

A total of 397 people filled out the questionnaire, 325 met the inclusion criteria after a trap question check (Table 3). 58.2% were married and 84.9% had a college/undergraduate degree or higher. The majority of them (79.7%) were willing to take the self‐funded test for BCS tests, and 22.5% of the participants had at least basic or more knowledge of breast cancer (Table 4).

**TABLE 3:** Response to fixed questions (*n* = 397).

Response	Option A (fixed question 1)	Option B (fixed question 1)	None (fixed question 1)
Option A (fixed question 2)	28	1	1
Option B (fixed question 2)	270	7	16
None (fixed question 2)	19	1	54

**TABLE 4:** Demographics and characteristics of patients in this study.

Characteristic	Level	Number (or mean) (%)
Number		325
Marriage (%)	Divorced	19 (5.8)
	Widowhood	1 (0.3)
	Unmarried	116 (35.7)
	Married	189 (58.2)
Education level (%)	University	244 (75.1)
	Primary	4 (1.2)
	Postgraduate	32 (9.8)
	Secondary	45 (13.8)
Income (%)	≤10,000元	87 (26.8)
	≥100,000元	15 (4.6)
	10,001–30,000元	147 (45.2)
	30001–50,000元	54 (16.6)
	50001–70,000元	14 (4.3)
	70,001–100,000元	8 (2.5)
Proactive screening (%)	Unwillingness	66 (20.3)
	Willingness	259 (79.7)
Knowledge about BSC (%)	Partially understanding	215 (66.2)
	Basic understanding	66 (20.3)
	Completely unknown	37 (11.4)
	Complete understanding	7 (2.2)

### 3.2 Importance of attributes and conditional logistic model

**FIGURE 1:**
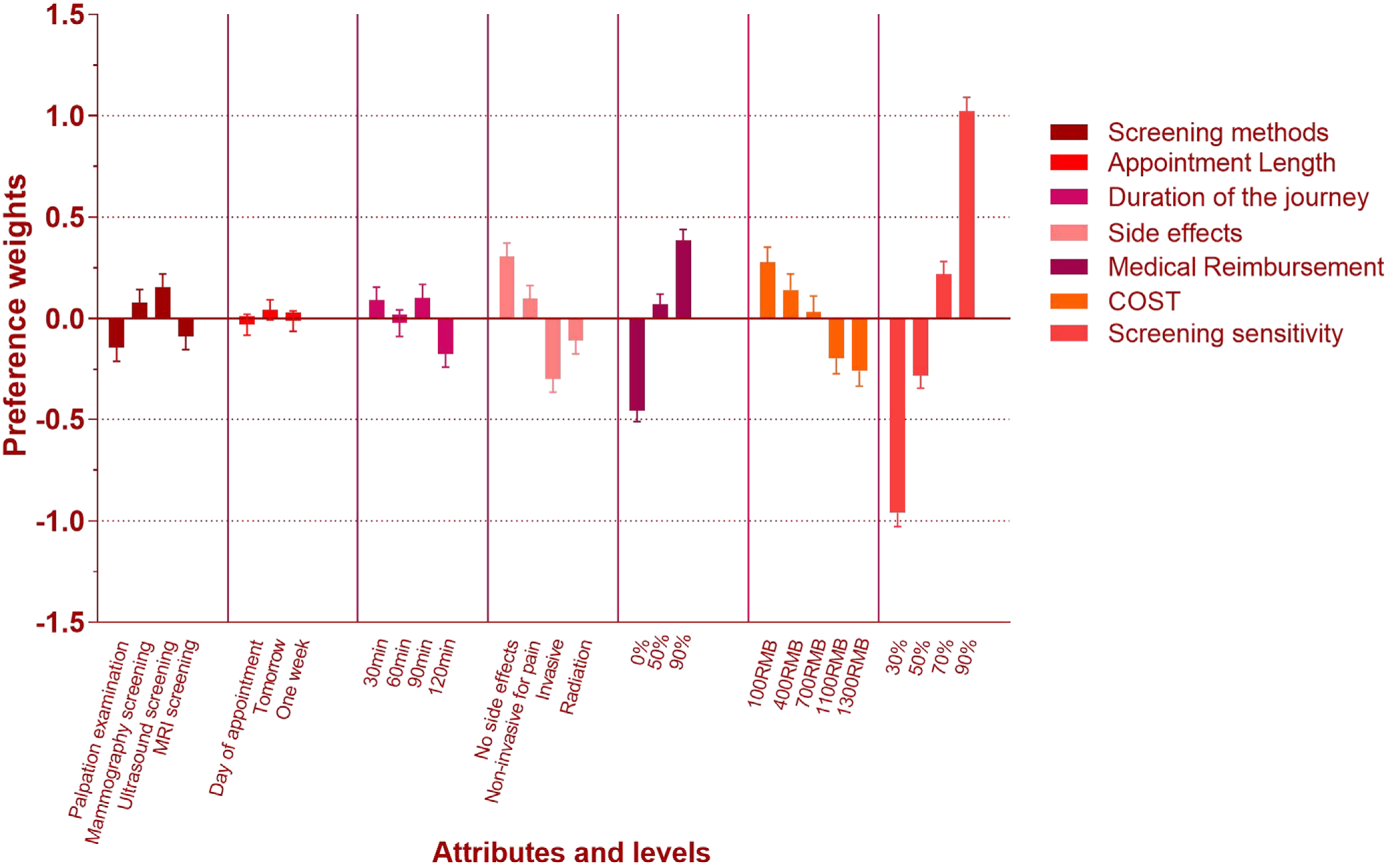
The visual presentation of estimated preference weights in the full sample.

**FIGURE 2:**
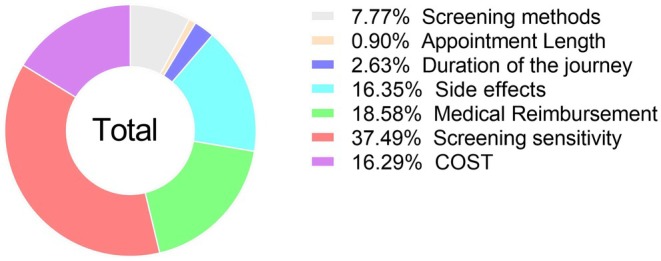
Percentage importance of attributes in the full sample.

Table 5 shows the result of the conditional logistic regression models. The significance of utilities indicates a significant impact on individual attributes. The sign of utilities represents the positive/negative impact of the attributes or levels on the consideration of the participants (Figure 1). As observed from Table 5, statistically significant differences (*p* < 0.05) were found in five out of seven attributes, namely screening tools, side effects, medical reimbursement, screening sensitivity, and cost. Overall, participants attach the most weight to the sensitivity of the screening test (Figure 2), accounting for 37.49%, followed by medical reimbursement (18.58%), potential risks (16.35%), costs (16.29%), and screening methods (7.77%).

**TABLE 5:** Estimated relative preference weights.

Attribute	Estimated preference weights		SE	*p*‐value
	Label	Utility		
Screening tools	Clinical breast examination	−0.19	0.05	<0.001
	Mammogram	0.11	0.05	0.017
	Ultrasonography	0.13	0.05	0.007
	MRI	−0.05	0.05	0.318
Appointment time	Day of appointment	0.02	0.04	0.571
	Tomorrow	−0.02	0.04	0.685
	1 week	−0.01	0.04	0.870
Travel time	30 min	0.03	0.05	0.553
	60 min	0.00	0.05	0.929
	90 min	0.04	0.05	0.425
	120 min	−0.07	0.05	0.146
Side effects	No	0.36	0.05	<0.001
	Pain	0.05	0.05	0.236
	Traumatic	−0.31	0.05	<0.001
	Radiation	−0.10	0.05	0.033
Medical reimbursement	0%	−0.39	0.04	<0.001
	50%	0.02	0.04	0.659
	90%	0.37	0.04	<0.001
Screening sensitivity	30%	−0.77	0.05	<0.001
	50%	−0.19	0.05	<0.001
	70%	0.22	0.05	<0.001
	90%	0.75	0.04	<0.001
COST	RMB￥100	0.39	0.05	<0.001
	RMB￥400	0.13	0.05	0.019
	RMB￥700	−0.02	0.05	0.728
	RMB￥1100	−0.22	0.06	<0.001
	RMB￥1400	−0.27	0.06	<0.001

Regarding the selection method, participant preferences for ultrasound screening and mammography (positive utility values) were compared from Figure 1 and Table 5. Clinical examination and MRI were disliked (negative utility values), but participants had an uncertain attitude toward MRI (*p* > 0.05). When choosing clinical examination as the reference level, the utility for ultrasound screening was 0.13 (*p* < 0.05), which was larger than the utility for mammography, which was 0.11 (*p* < 0.05). This suggests that participants were more inclined toward ultrasound screening in their preference for screening tools.

Concerning side effects, participant preferences were positive for no side effects and pain (positive utility values) and negative for trauma and radiation (negative utility values). However, the utility value for pain was lower and not statistically different, indicating uncertain attitudes toward pain. When using no side effects as the reference level, the utility for radiation was −0.10 (*p* < 0.05), which was lower than the utility for trauma, which was −0.31 (*p* < 0.05). This suggests that participants disliked trauma more than radiation.

Regarding medical reimbursement, participant preferences were higher for higher reimbursement percentages, such as 90% (positive utility value), and negative for a low reimbursement percentage of 0% (negative utility value), with 50% reimbursement being the threshold of hesitation. When using 0% reimbursement as the reference level, the utility for 90% reimbursement had the highest value at 0.37 (*p* < 0.05), indicating a preference for 90% reimbursement compared to 0%.

Concerning screening sensitivity, participants preferred higher sensitivity (positive utility value), with a minimum threshold of >50%. When using 30% sensitivity as the reference level, the utility for 90% sensitivity had the highest value at 0.75 (*p* < 0.05), which was higher than the values for 50% and 70%. This indicates a preference for 90% screening sensitivity over 30%.

Regarding costs, participants preferred lower costs, such as RMB100 (positive utility value), and disliked higher costs, such as RMB1400 (negative utility value). It is worth noting that RMB700 might be the threshold of hesitation for participants (*p* > 0.05).

While Figure 1 demonstrates positive or negative utility values for appointment time and travel time, the analysis in conjunction with the results from Table 5 revealed no statistically significant differences (*p* > 0.05). Further research is required to analyze participants' preferences regarding these attributes.

### 3.3 Willingness to pay

Table 6 indicates that participants favor “Ultrasound Screening” and “no adverse impact.” They are willing to spend CNY 572.30 for ultrasound screening instead of palpation inspection. Moreover, they are willing to spend CNY 25.00 and CNY 313.14 respectively to upgrade their screening from mammogram and MRI test to ultrasound screening. Respondents strongly prefer nonadverse impact tests and are willing to pay up to CNY 1204.52 for a noninvasive test. They are willing to pay CNY 829.36 to avoid a screening test with radiation and CNY 1.95 per minute to reduce waiting time. Importantly, they are also willing to pay CNY 46.04 to maintain a 1% sensitivity.

**TABLE 6:** Willingness to pay.

Attribute	Utility difference	Willingness to pay	Willingness to pay
		RMB (￥)	RMB (￥)
Screening method (CBE)		Reference	
Screening method (mammogram)	0.30	−547.30	−25.00
Screening method (ultrasonography)	0.32	−572.30	Reference
Screening method (MRI)	0.14	−259.16	−313.14
Appointment time (day of appointment)		Reference	
Appointment time (1 day)	−0.04	66.21	
Appointment time (1 week)	−0.03	49.86	
Travel time (per 1% reduction)	−0.00	1.95	
Side effects (none)		Reference	
Side effects (pain)	−0.30	544.87	
Side effects (traumatic)	−0.66	1204.52	
Side effects (radiation)	−0.46	829.36	
Medical reimbursement (per 1% increase)	0.01	−15.21	
Screening sensitivity (per 1% increase)	0.03	−46.04	
Test cost	−0.00	Reference	

### 3.4 Latent class model

**FIGURE 3:**
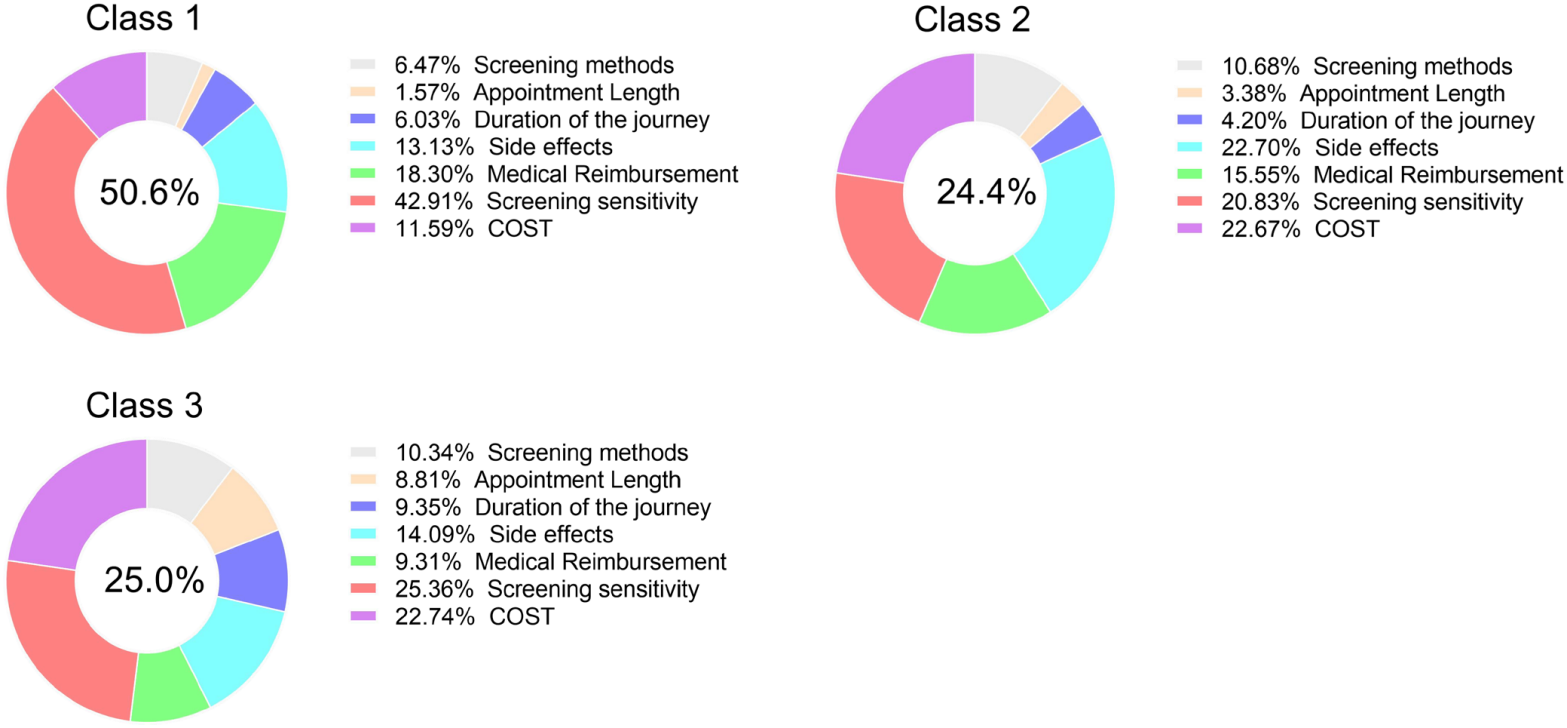
Percentage importance of attributes in the latent class condition.

There are three potential categories for this experiment based on the Bayesian standard minimum. All 325 respondents were classified into three categories: 164 (50.6%), 79 (24.4%), and 82 (25%). The mean maximum membership was 0.96 and the percentage certainty was 39.72. For the first class, the attribute “screening sensitivity” was the most important attribute for respondents, with a percentage importance value of 42.91%, followed by “medical insurance reimbursement” and “side effects” with percentage importance values of 18.3% and 13.13% (Figure 3).

For the second class, the attributes “cost” and “side effects” were relatively important, with percentage importance values of 22.67% and 22.70% respectively for this class of respondents. For the attribute “screening sensitivity,” the percentage importance was 20.83% (Figure 3).

For the third class, the attributes “screening sensitivity” and “cost” were the most important factors considered by respondents, with a percentage importance ratio of 25.36% and 22.74%, respectively (Figure 3). The next most important attribute was “side effects,” with a percentage importance of 14.09% (Figure 3).

For these three potential classes, the attributes “side effects,” “medical reimbursement,” “screening sensitivity,” and “cost” were the top considerations for respondents (Figure 3), which was consistent with the analysis of Table 7.

**TABLE 7:** Estimated relative preference weights for the three classes.

Attributes and levels	Class 1 (*n* = 164)	*p* value	Class 2 (*n* = 79)	*p* value	Class 3 (*n* = 81)	*p* value
	Coefficient (SE)		Coefficient (SE)		Coefficient (SE)	
Screening methods						
Clinical breast examination	−0.14 (0.07)	0.029	−0.43 (0.11)	<0.001	−0.65 (0.29)	0.025
Mammography screening	0.078 (0.06)	0.228	0.28 (0.10)	0.007	0.01 (0.24)	0.973
Ultrasound screening	0.15 (0.06)	0.017	0.20 (0.10)	0.056	0.26 (0.22)	0.246
MRI screening	−0.09 (0.06)	0.165	−0.05 (0.11)	0.664	0.38 (0.22)	0.086
Appointment length						
Day of appointment	−0.03 (0.05)	0.555	0.14 (0.08)	0.102	0.40 (0.19)	0.030
Tomorrow	0.04 (0.05)	0.399	−0.09 (0.09)	0.314	−0.48 (0.22)	0.028
1 week	−0.01 (0.05)	0.806	−0.05 (0.08)	0.547	0.07 (0.19)	0.704
Travel time						
30 min	0.09 (0.06)	0.140	−0.11 (0.11)	0.302	0.24 (0.23)	0.290
60 min	−0.02 (0.06)	0.724	0.05 (0.10)	0.633	0.03 (0.25)	0.895
90 min	0.10 (0.06)	0.106	−0.10 (0.11)	0.336	0.33 (0.22)	0.133
120 min	−0.17 (0.06)	0.007	0.17 (0.10)	0.112	−0.61 (0.29)	0.035
Side effects						
No side effects	0.31 (0.07)	<0.001	0.74 (0.10)	<0.001	0.83 (0.21)	<0.001
Noninvasive for pain	0.10 (0.06)	0.121	0.01 (0.11)	0.917	0.17 (0.23)	0.454
Invasive	−0.30 (0.06)	<0.001	−0.76 (0.12)	<0.001	−0.58 (0.30)	0.053
Radiation	−0.11 (0.06)	0.094	0.00 (0.10)	0.997	−0.42 (0.28)	0.130
Medical Reimbursement						
0%	−0.46 (0.05)	<0.001	−0.42 (0.09)	<0.001	−0.38 (0.22)	0.083
50%	0.07 (0.05)	0.162	−0.18 (0.09)	0.032	−0.18 (0.21)	0.386
90%	0.39 (0.05)	<0.001	0.61 (0.08)	<0.001	0.56 (0.18)	0.003
Screening sensitivity						
30%	−0.96 (0.07)	<0.001	−0.67 (0.11)	<0.001	−1.02 (0.41)	0.013
50%	−0.28 (0.06)	<0.001	−0.26 (0.11)	0.015	−0.72 (0.35)	0.041
70%	0.22 (0.06)	<0.001	0.22 (0.10)	0.032	0.23 (0.28)	0.405
90%	1.02 (0.07)	<0.001	0.71 (0.10)	<0.001	1.52 (0.23)	<0.001
Total cost of screening						
100RMB	0.28 (0.08)	<0.001	0.91 (0.11)	<0.001	1.03 (0.24)	<0.001
400RMB	0.14 (0.08)	0.069	0.35 (0.12)	0.004	0.27 (0.28)	0.326
700RMB	0.03 (0.08)	0.669	−0.10 (0.12)	0.400	−0.03 (0.29)	0.914
1100RMB	−0.20 (0.08)	0.011	−0.56 (0.13)	<0.001	−0.02 (0.30)	0.941
1300RMB	−0.26 (0.08)	0.001	−0.59 (0.13)	<0.001	−1.25 (0.47)	0.009

**FIGURE 4:**
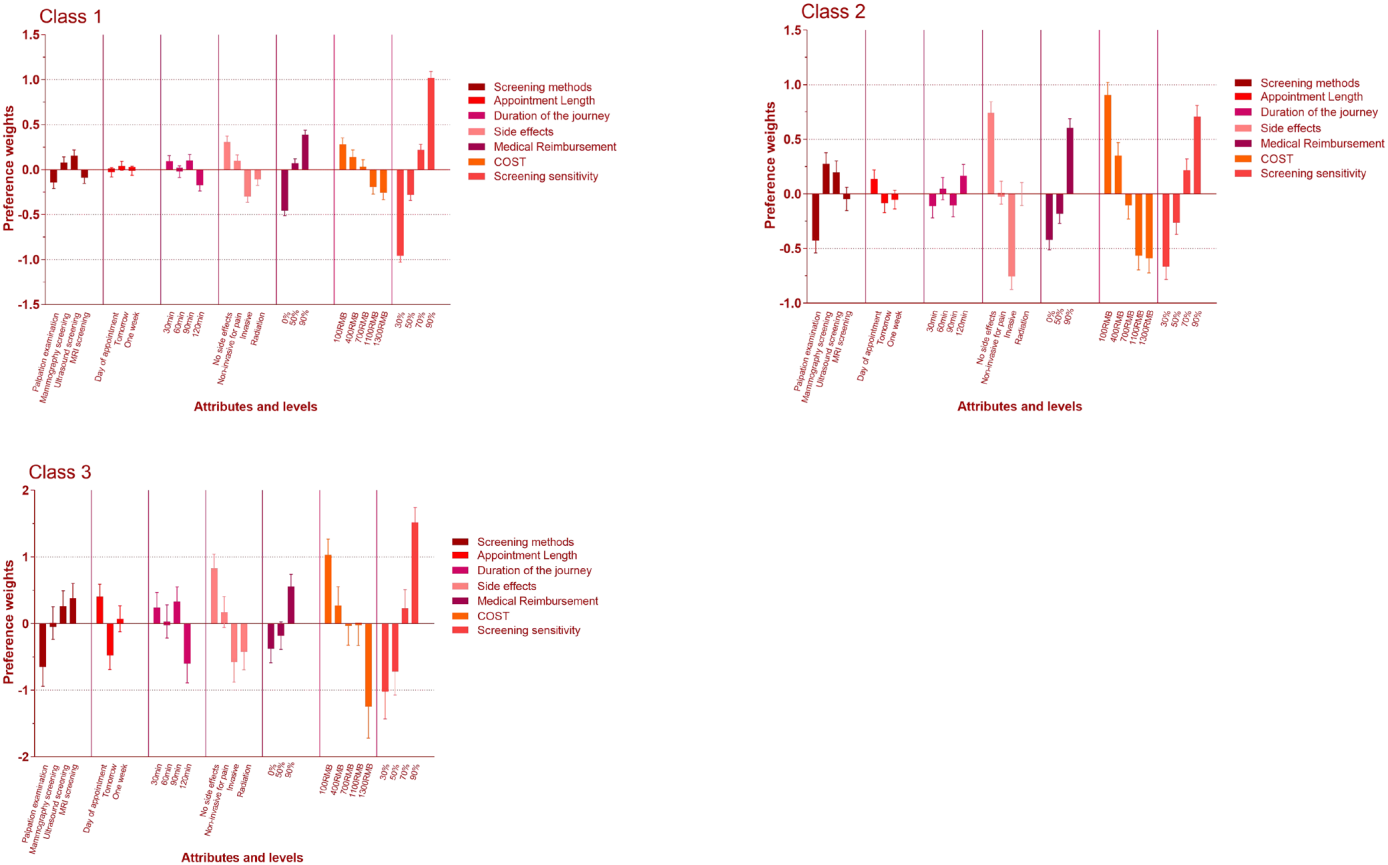
The visual presentation of estimated preference weights in the latent class condition.

The utility sign serves as an indicator of the directional influence (positive or negative) that attributes or levels exert on the evaluation of estimated preference weights under the framework of latent class analysis (as depicted in Figure 4).

## 4 DISCUSSION

The current study is the first DCE study to explore Chinese women's preferences for BCS in China. This study examined Chinese women's BCS preferences using quantitative methods. We found that participants have significant preference for high screening sensitivity and high reimbursement of Medicare. Compared to other attributes, participants did not care as much about travel times, appointment times, and screening tools. We also found that participants were not willing to undertake the high costs and accept clinical breast examinations. This study explicitly quantified the weighting of each attribute of BC screening that the individual was willing to receive through the DCE, filling the research gap in this area in China.

Women in our study were averse to clinical breast examinations compared to a DCE study in BCS in Belarus,^15^ where women in both studies emphasized higher sensitivity, Medicare reimbursement, and cost in screening. The possibility of explaining this difference is that clinical breast examinations are less sensitive and usually more specific.^16^ Moreover, the accuracy of clinical breast examination is also related to the experience of the doctor (Figure 4). Another explanation for this difference is the traditional mindset of Eastern women, which differs from that of Western women. Oriental women are reluctant to expose their bodies to staff during clinical breast examinations.^17^

In other disease screening DCE studies, there is a tendency to prefer shorter travel times.^18^ In our study on BCS, we observed that participants did not place a high emphasis on shorter waiting times for medical appointments. Unlike general healthcare‐seeking behaviors, participants expressed a lack of concern regarding waiting times ranging from 1 to 4 h for travel time and even waiting for a week for their screening. This finding deviates from the typical expectations associated with seeking medical care. In this discussion, we explore potential reasons for this phenomenon, focusing on the absence of symptoms, longer screening intervals, and the psychological well‐being of participants.

### 4.1 Absence of symptoms and longer screening intervals

One plausible explanation for participants' indifference toward shorter waiting times is the absence of symptoms. BCS predominantly targets asymptomatic individuals who are not experiencing any noticeable physical discomfort or signs of illness. This lack of symptoms may lead participants to perceive screening as a less urgent matter compared to seeking care for acute conditions. Consequently, they may be more accepting of longer waiting times, considering that the absence of symptoms implies a lower immediate health risk.

Moreover, the longer screening intervals implemented in BCS programs could contribute to participants' perception of acceptable waiting times. Screening guidelines often recommend periodic screenings, such as annually or every 2 years. As a result, participants may perceive waiting for an extended period as reasonable, as it aligns with the recommended screening intervals. This perception further diminishes the sense of urgency for immediate appointments and shorter waiting times.

### 4.2 Psychological factors

An additional factor influencing participants' preferences for longer waiting times in BCS could be attributed to their psychological well‐being. Participants may exhibit a positive psychological state, characterized by a lack of worry, fear, or anxiety related to BCS. This psychological state may result from their confidence in the effectiveness of screening procedures, trust in the healthcare system, or a general sense of well‐being. In the absence of significant psychological distress or concerns, participants may not perceive waiting times as burdensome or anxiety‐inducing, hence their indifference toward shorter waiting periods.

Because women are most concerned about the sensitivity and cost of screening, medical staff should provide women with detailed information on the detection rates and costs of each screening.

Furthermore, our research indicated that the highest priority screening should be sensitive and not cause discomfort. There is no optimal screening for breast cancer, although some experts suggest that mammograms can be combined with ultrasound, the accuracy of which is dependent on the skill level and experience of the examiner. New screening methods are currently being developed, such as biological criteria for the early detection of breast cancer through nipple secretions.^19^ The results of this DCE can be used to guide its use in future BCS.

The study findings could be used to guide the potential inclusion of breast cancer coverage in medical insurance and assist doctors in understanding the factors that influence women's decisions regarding BCS. This, in turn, would enable them to offer appropriate screening methods.

### 4.3 Limitations

While our study utilized a rigorous DCE approach for designing the questionnaire and analyzing the results, there are some limitations. First, it is important to note that our sample may not fully represent the diversity of women in China, as participants were recruited from various community hospitals in the Nanshan District of Shenzhen. Second, it is worth mentioning that participants had difficulty accepting that the attribute levels in the options did not align with their perception of reality, which could introduce a potential bias in their responses. Last, it is also worth considering that demographic factors, such as occupation, education level, and income, may also influence women's preferences for BCS. Further analysis of these demographic characteristics is required to gain a better understanding of preferences among different population groups.

## 5 CONCLUSION

Chinese women tend to prefer machine‐assisted inspection rather than doctor palpation for BCS. The findings of this study also assist in prioritizing women's preferences for screening attributes and provide guidance for enhancing the screening system at the grassroots level.

## AUTHOR CONTRIBUTIONS

**Shuning Wu:** Conceptualization (equal); data curation (equal); formal analysis (equal); investigation (equal); methodology (supporting); visualization (equal); writing – original draft (lead); writing – review and editing (supporting). **Jianhao Du:** Conceptualization (supporting); data curation (lead); formal analysis (equal); investigation (equal); methodology (supporting); visualization (equal); writing – original draft (equal); writing – review and editing (supporting). **Yifei Wang:** Data curation (equal); formal analysis (supporting); investigation (supporting); visualization (supporting); writing – original draft (supporting); writing – review and editing (supporting). **Dawei Peng:** Data curation (supporting); formal analysis (supporting); investigation (supporting); visualization (supporting); writing – original draft (supporting); writing – review and editing (supporting). **Xinyang Ma:** Data curation (supporting); formal analysis (supporting); investigation (supporting); writing – original draft (supporting); writing – review and editing (supporting). **Jiali Chen:** Data curation (supporting); investigation (supporting); writing – original draft (supporting); writing – review and editing (supporting). **Jie Li:** Data curation (supporting); investigation (supporting); writing – original draft (supporting); writing – review and editing (supporting). **Gezi Li:** Data curation (supporting); investigation (supporting); writing – original draft (supporting); writing – review and editing (supporting). **Xing Li:** Data curation (supporting); investigation (supporting); writing – original draft (supporting); writing – review and editing (supporting). **Jun Zhang:** Conceptualization (equal); formal analysis (equal); methodology (equal); project administration (equal); supervision (equal); validation (equal); visualization (equal); writing – original draft (equal); writing – review and editing (equal). **Wai‐Kit Ming:** Conceptualization (lead); formal analysis (lead); investigation (lead); methodology (lead); project administration (lead); supervision (lead); validation (lead); visualization (lead); writing – original draft (equal); writing – review and editing (lead).

## FUNDING INFORMATION

This research was partially supported by SIRG—CityU Strategic Interdisciplinary Research Grant (no. 7020093).

## CONFLICT OF INTEREST STATEMENT

The authors declare that they have no competing interests.

## ETHICS APPROVAL AND CONSENT TO PARTICIPATE

The present study was conducted after the approval of the Institutional Review Board of Shenzhen Qianhai Shekou Free Trade Zone Hospital and adhered to the guidelines outlined in the Declaration of Helsinki. All participant data were anonymized, and informed consent was obtained.

## CONSENT FOR PUBLICATION

Not applicable.

## Data Availability

The datasets used and/or analyzed during the current study are available from the corresponding author on reasonable request.
